# Downstream targets of methyl CpG binding protein 2 and their abnormal expression in the frontal cortex of the human Rett syndrome brain

**DOI:** 10.1186/1471-2202-11-53

**Published:** 2010-04-26

**Authors:** Joanne H Gibson, Barry Slobedman, Harikrishnan KN, Sarah L Williamson, Dimitri Minchenko, Assam El-Osta, Joshua L Stern, John Christodoulou

**Affiliations:** 1 Western Sydney Genetics Program, The Children's Hospital at Westmead, Sydney, Australia; 2 Discipline of Pediatrics and Child Health, University of Sydney, Sydney, Australia; 3 Centre for Virus Research, Westmead Millennium Institute, Sydney, Australia; 4 Alfred Medical Research and Education Precinct (AMREP), Baker IDI Heart and Diabetes Institute, Melbourne, Australia; 5 Institute of Endocrinology and Diabetes, The Children's Hospital at Westmead, Sydney, Australia; 6 Current address: The Picower Institute of Learning and Memory, Massachusetts Institute of Technology, Cambridge, Massachusetts, 02144, USA

## Abstract

**Background:**

The Rett Syndrome (RTT) brain displays regional histopathology and volumetric reduction, with frontal cortex showing such abnormalities, whereas the occipital cortex is relatively less affected.

**Results:**

Using microarrays and quantitative PCR, the mRNA expression profiles of these two neuroanatomical regions were compared in postmortem brain tissue from RTT patients and normal controls. A subset of genes was differentially expressed in the frontal cortex of RTT brains, some of which are known to be associated with neurological disorders (*clusterin *and *cytochrome c oxidase subunit 1*) or are involved in synaptic vesicle cycling (*dynamin 1*). RNAi-mediated knockdown of MeCP2 *in vitro*, followed by further expression analysis demonstrated that the same direction of abnormal expression was recapitulated with MeCP2 knockdown, which for *cytochrome c oxidase subunit 1 *was associated with a functional respiratory chain defect. Chromatin immunoprecipitation (ChIP) analysis showed that MeCP2 associated with the promoter regions of some of these genes suggesting that loss of MeCP2 function may be responsible for their overexpression.

**Conclusions:**

This study has shed more light on the subset of aberrantly expressed genes that result from *MECP2 *mutations. The mitochondrion has long been implicated in the pathogenesis of RTT, however it has not been at the forefront of RTT research interest since the discovery of *MECP*2 mutations. The functional consequence of the underexpression of *cytochrome c oxidase subunit 1 *indicates that this is an area that should be revisited.

## Background

Rett syndrome (RTT) is a neurodevelopmental disorder and a leading cause of severe intellectual disability in females [1]. Distinguishing features of RTT include a loss of previously acquired skills such as communication, purposeful hand movements and mobility, as well as deceleration of head growth, seizures, and the development of characteristic stereotypic hand wringing.

One of the most prominent neuropathological features of RTT is the impairment of normal neuronal development, primarily at the synapse with a disruption to the normal development of axodendritic connections. There is a decrease in the size of the RTT brain which is partially explained by a decrease in the size of individual neuronal cell bodies, an increase in the packing density of neurons [2], possibly an absence of specific neuronal populations [3] and a decrease in dendritic arborization [4]. Additionally, areas of 'naked' dendritic spines are observed on pyramidal neurons of layer II and III in the frontal cortex [5].

The RTT neuropathology manifests in specific regions of the brain. The shortening and thickening of dendritic branches are observed in the same brain regions that have a decreased volume, for example layers III and V of the frontal, motor and inferior temporal regions, whereas the dendrites of the occipital cortex are comparatively less affected [4].

A diagnosis of RTT is typically made on the evolving clinical phenotype, although most cases have a genetic basis and are caused by mutations in the X-linked *MECP2 *gene (Methyl-CpG Binding Protein 2) [6]. MeCP2 has been thought to be a transcriptional repressor that acts by binding to methylated CG dinucleotides in some gene promoters, ultimately causing chromatin compaction leading to gene silencing. More recent studies suggest that it may also mediate splicing [7], may be involved in the higher order organization of chromatin architecture [8], and in some circumstances can act as a transcriptional activator [9].

A small subset of atypical cases with a RTT-like phenotype, characterized by the early onset of seizures, is associated with mutations in *CDKL*5 (Cyclin-Dependent Kinase-like 5) [10-12], whilst some individuals with the congenital RTT variant have mutations in the *FOXG1 *(forkhead box protein G1) gene [13], and still others remain genetically undefined.

The localization of MeCP2 to the postsynaptic region [14] as well as the nucleus, coupled with the possibility that more than one gene locus is affected, suggests that dysfunction of more than one molecular pathway may contribute to the neurological phenotype, and these pathways could involve abnormal transcription and/or synaptic transmission.

In order to gain an insight into the molecular pathways that may be affected in RTT, it is important to determine the genes whose transcription patterns are altered. Recent studies have uncovered a number of these genes in frontal cortices of human [15], human non-clonal [16] and clonal [17] lymphoblast cell lines, human clonal fibroblasts [18], and various *Mecp2 *mouse models [9,19-22]. Some genes have been found to be under the direct transcriptional control of MeCP2 and so the downstream effect of this misregulation offers clues as to the molecular basis of the severe neurological deficit that is observed in RTT.

In this study we capitalized on the observation that RTT pathology is predominates in specific neuroanatomical regions of the brain. cDNA microarray analysis uncovered a number of genes that were abnormally expressed in the frontal cortex, but not in the occipital cortex of RTT patients. Using RNA interference, the differential expression of these genes was confirmed *in vitro *to result from a deficiency in MeCP2. The direct interaction between MeCP2 and the promoter of some of these genes was confirmed by chromatin immunoprecipitation (ChIP). In addition, the functional consequence of the differential expression of one of the genes, *cytochrome c oxidase subunit 1*, was evaluated.

## Results

### Differentially expressed genes in the frontal cortex of Rett syndrome brains

Control brain samples were (unfortunately) not age-matched (age range 31 - 52 yr; age range of RTT patients 4 - 21 yr). At the time the study was performed, we were unable to access age-matched controls from Australia or the USA. We acknowledge that expression patterns may change with age, however, the genes that were analyzed following initial microarray analysis were also (1) differentially expressed in an *in vitro *RNAi-mediated knockdown system and (2) differentially expressed between the frontal and occipital cortex of the individual RTT brain samples, independent of any comparison with the control brain samples. It should be noted that after careful consideration it was decided that this was a unique opportunity to be able to utilize these postmortem samples that we had available to us, however it was obvious to us from the outset that using postmortem brain samples of different ages, was a shortcoming of the study.

**Figure 1:**
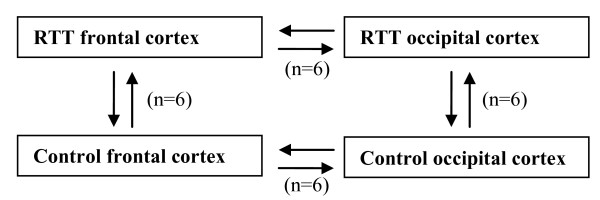
**Microarray experimental design**. Four sets of six microarray experiments were conducted. The double arrows represent co-hybridization of the samples at each end of the arrows. Six individual RTT frontal cortices were co-hybridized with six individual control frontal cortices. For example, the frontal cortex of patient RTT1 was compared with the occipital cortex of RTT1. Additionally, where the frontal cortex of a RTT sample was compared to the frontal cortex of a control sample, the comparison between the occipital cortices was conducted between the same samples. For example RTT1 frontal cortex *vs. *Con4 frontal cortex, RTT1 occipital cortex *vs. *Con4 occipital cortex. Each experiment was repeated and the fluorescent cyanine dyes were reversed.

The frontal cortex is one of the brain regions in RTT that displays the characteristic histopathology associated with this disorder. In order to establish if there is abnormal expression of a subset of genes in the RTT frontal cortex, we examined and compared mRNA expression profiles between the RTT and control frontal and occipital cortices. Four sets of six microarray experiments were conducted (Figure 1). The double arrows represent co-hybridization of the samples at each end of the arrows. For example, six individual RTT frontal cortices were co-hybridized with six individual control frontal cortices.

Based on this experimental design differential expression between: (a) the RTT frontal cortex and control frontal cortex would possibly be due to individual variation or could be contributing to RTT pathology; (b) the RTT frontal cortex and RTT occipital cortex would possibly be due to normal expression differences that occur between these brain regions or could be contributing to RTT pathology, and (c) the control frontal cortex and control occipital cortex would possibly be due to normal expression differences that occur between these brain regions. Using a subtractive method, genes that were exclusively differentially expressed in the RTT frontal cortex were determined as those that were differentially expressed in (a) and (b), but not in (c). Genes that were differentially expressed between the RTT occipital cortex and control occipital cortex were considered to be due mostly to individual variation as this region appears to be spared the reduction in dendritic trees [4] and volume that is observed in other regions of the RTT brain.

Samples were evaluated on an individual basis, RNA samples were not pooled.

The number of transcripts that reached 'cut-off' for each set of analyses were those that were expressed above minimal pixel intensity, and were therefore considered to be expressed in the brain samples analyzed (Table 1). Those that were considered differentially expressed either by 'fold-change' analysis or by SAM analysis were determined and the genes of interest in the group that overlapped these two types of analysis are listed in Table 2. The expression of these genes was verified using quantitative reverse transcription (qRT)-PCR.

**Table 1:** Summary of transcripts showing differential expression.

Comparison	Number of transcripts that passed 'cut-off'	Number of differentially expressed transcripts		
		**Fold-change**	**SAM analysis**	**Overlap**

RTT fc ***vs. ***CON fc	11,812	34	891	15

RTT fc ***vs. ***RTT oc	9,334	46	13	9

RTT oc ***vs. ***CON oc	9,355	21	0	-

CON fc ***vs. ***CON oc	10,087	13	19	4

The number of genes that passed cut-off were those that were expressed (pixel intensity of greater than 200 in one channel) and undistorted (not 'flagged') in each group of 6 experiments. The number of genes that was deemed significantly differentially expressed is listed for the 'fold change' (1.5 fold change) and the SAM analysis comparison. fc = frontal cortex; oc = occipital cortex. A gene that was differentially expressed between RTT fc ***vs. ***CON fc AND RTT fc ***vs. ***RTT oc, and not in the CON fc ***vs. ***CON oc was considered for further analysis. Column labeled 'overlap' contains the number of transcripts that were differentially expressed in both the fold-change and the SAM analysis.

**Table 2:** Differentially expressed genes in the RTT frontal cortex (microarray analysis).

GENBANK ACC #	GENE NAME	GENE SYMBOL	CHR	FUNCTION	UP OR DOWN REGULATED (average of comparisons)
H14069	Amyloid beta (A4) precursor-like protein 1	*APLP1*	19q13.1	Enhancer of neuronal apoptosis	UP 2.0 SD 0.47 SEM 0.24

W68191	Clusterin (apolipoprotein J)	*CLU*	8p21-p12	Extracellular molecular chaperone	UP 1.6 SD 0.12 SEM 0.06

H30899	Collapsin response mediator protein 1	*CRMP1*	4p16.1-p15	Part of semaphorin signal transduction pathway	UP 2.6 SD 0.14 SEM 0.08

BE879779 BM888296 BM842146	Cytochrome c oxidase subunit I	*CO1*	M	Mitochondrial respiratory chain	DOWN 3.3 SD 0.61 SEM 0.27

H51542	Dynamin I	*DNMI*	9q34	Produces microtubule bundles. Binds and hydrolyzes GTP. Vesicular trafficking	UP 2.0 SD 0.63 SEM 0.31

BE513417	Echinoderm microtubule associated protein like 2 (*Homo sapiens*)	*EML2*	19q13.32	Modifies the assembly dynamics of microtubules	DOWN 1.9 SD 0.05 SEM 0.02

AL565619	GDP dissociation inhibitor 1	*GDI1*	Xq28	Regulates the GDP/GTP exchange	UP 1.78 SD 0.33 SEM 0.17

H14897	Guanine nucleotide binding protein (G protein), beta polypeptide 1	*GNBI*	1p36.33	Integrates signals between receptors and effector proteins	UP 2.5 SD 0.90 SEM 0.45

BG167084 BM783962	16S ribosomal RNA/Humanin	*HMN*	M*	Potential role in neurons via a putative cell-surface receptor through which it exhibits neuroprotective activity	UP 2.1 SD 0.34 SEM 0.17

R52851	Reticulon 3	*RTN3*	11q13	Blocks access of BACE1 (Beta-site APP-cleaving enzyme 1) to APP (amyloid precursor protein) within neurons	UP 2.5 SD 0.82 SEM 0.41

These genes were differentially expressed in the RTT frontal cortex when compared to expression in the RTT occipital cortex and the control frontal cortex. N.B. The value given for differential expression (either UP or DOWN) refers to the average of the experiments; SD = standard deviation; SEM = standard error of the mean; M = mitochondrial.

* This sequence is 99% identical to 16S ribosomal mitochondrial RNA, and also to some nuclear encoded cDNAs.

**Figure 2:**
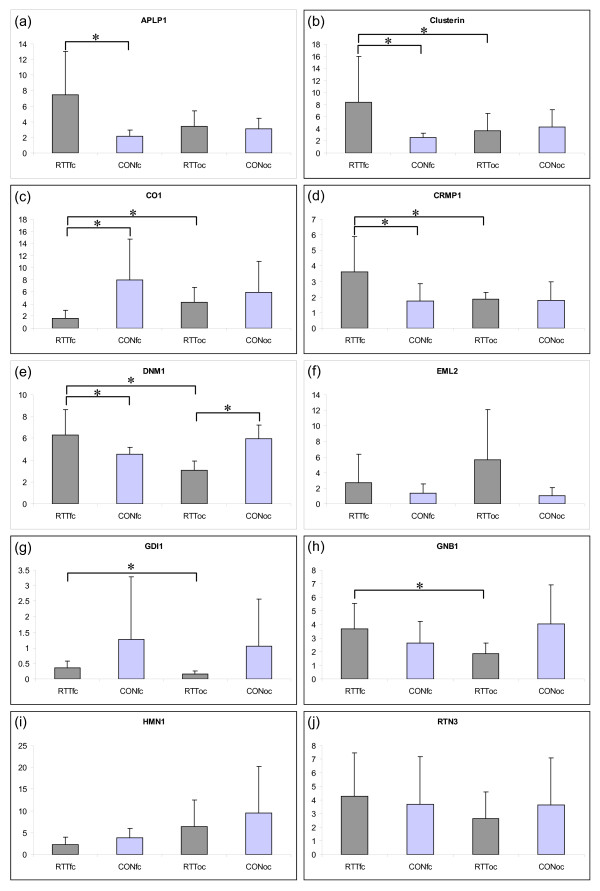
**Expression of genes of interest in RTT and control samples, individual samples**. The expression of genes of interest was measured by quantitative RT-PCR. Expression of each transcript was evaluated in six RTT and six control frontal and occipital cortices, and normalized to the expression of *GAPDH *in the same sample. **RTT **= Rett syndrome; **CTL **= control; **FC **= frontal cortex and **OC **= occipital cortex. Samples that were expressed more than 10-fold higher than housekeeping genes were serially diluted and multiple dilutions were analyzed. The Y-axis shows the ratio of the transcript of interest normalized against *GAPDH ***(a) *APLP1 ***mRNA expression levels in RTT FC were significantly higher than in control FC (p = 0.02), but not compared to RTT OC (p = 0.06); **(b) *CLU ***mRNA expression levels in RTT FC were significantly higher than in control FC (p = 0.042) and RTT OC (p = 0.047); **(c) *CO1 ***mRNA expression levels in RTT FC were significantly lower than in control FC (p = 0.024) and RTT OC (p = 0.024); **(d) *CRMP1 ***mRNA expression levels in RTT FC were significantly higher than in control FC (p = 0.049) and RTT OC (p = 0.045); **(e) *DNMI ***mRNA expression levels in RTT FC were significantly greater than in the control frontal cortex (p = 0.048) and RTT OC (p = 0.004), however expression in the RTT OC was significantly lower than in the control OC (p = 0.004); **(f) *EML2 ***mRNA expression levels in RTT FC were not significantly different to the RTT OC (p = 0.062), or the control FC (p = 0.213); **(g) *GDI1 ***expression in the RTT FC was significantly greater than the RTT OC (p = 0.045), but not compared to expression in the control FC (p = 0.14); **(h) *GNBI ***mRNA expression in the RTT FC was significantly greater than in the RTT OC (p = 0.026), but not compared to the control OC (p = 0.164); **(i) *HUM ***mRNA expression in the RTT FC was not significantly greater than in the RTT OC (0.064) or the control FC (p = 0.092); **(j) *RTN3 ***mRNA expression in the RTT FC was not significantly greater than in the RTT OC (p = 0.148) or the control FC (p = 0.378).

qRT-PCR was performed using new RNA extractions taken from the same tissue as that subjected to microarray analysis. *CLU*, *CO1, CRMP1 *and *DNM1 *were each differentially expressed in the RTT frontal cortex when compared to the RTT occipital cortex (p = 0.047, 0.024, 0.045 and 0.004, respectively) AND when compared to the control frontal cortex (p = 0.042, 0.024, 0.049 and 0.048, respectively; Figure 2b, c, d and 2e). Each was differentially expressed in the same direction as in the microarray analysis. Interestingly, *DNM1 *was upregulated in the RTT frontal cortex compared to the RTT occipital cortex (p = 0.004), and compared to the control frontal cortex (p = 0.048) however, the expression in the RTT occipital cortex was also significantly lower than the control occipital cortex (p = 0.015), suggesting that it was downregulated in the RTT occipital cortex. *APLP1 *was significantly upregulated in the RTT frontal cortex compared to the control frontal cortex (p = 0.020). Both *GNB1 *and *GDI1 *were significantly differentially expressed in the RTT frontal cortex compared to the RTT occipital cortex (p = 0.026 and 0.045, respectively). *GDI1 *appeared to be expressed less in the RTT frontal cortex compared to the control frontal cortex however this was not statistically significant. *EML2*, *HMN1 *and *RTN3 *were not significantly differentially expressed by qRT-PCR, even though *EML2 *and *RTN3 *showed an expression trend similar to that observed in the microarray experiments.

These data indicate that a subset of genes is (on average) differentially expressed in the frontal cortex of RTT patients. In summary, *CLU*, *CO1*, *CRMP1 *and *DNM1 *were specifically differentially expressed in the RTT frontal cortex compared to RTT occipital cortex and control frontal cortex, *APLP1 *was specifically differentially expressed in the RTT frontal cortex compared to the control frontal cortex and *GDI1 *and *GNB1 *were differentially expressed in the RTT frontal cortex when compared to the RTT occipital cortex. *EML2*, *HMN1 *and *RTN3 *were not differentially expressed.

### DNMI, GNBI, CO1 and CLU are differentially expressed following *in vitro* RNAi-mediated MeCP2 knockdown

**Figure 3:**
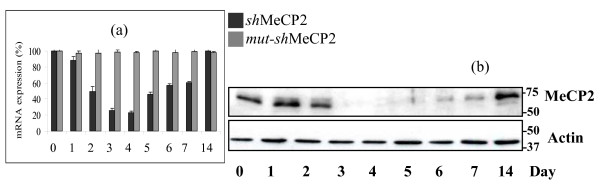
**RNAi-mediated knockdown of MECP2**. *MECP2*/MECP2 expression following RNAi-induced MeCP2 (e1 and e2) knockdown. Cells were harvested at time points over a 2-week period. (a) *MECP2 *mRNA expression in SH-SY5Y cells. Transcript levels were assessed by Real-Time qPCR and compared to the expression of *GAPDH *in each sample. *MECP2 *expression was quantitated at each time point, and presented as a percentage of the expression level at day 0, which was set at 100%. The knockdown sample (*sh*-MECP2) displays a 75-80% reduction in *MECP2 *expression when compared to the negative control (*mut-sh*MECP2) or to untreated cells (data not shown). *MECP2 *mRNA expression in the negative control remained above 95% greater than all time points; (b) MECP2 protein expression is reduced by 90% at Day 3. Each protein sample was divided in two and loaded onto duplicate gels, one immunoblotted with α-MECP2 and the other with α-actin.

To provide more specific evidence that reduced expression or function of MeCP2 is responsible for the altered mRNA expression noted in the microarray experiments, RNAi was used to knockdown MeCP2 *in vitro*. A 21 base pair dsRNA construct that targeted exon 3 of *MECP2 *was transfected into cultured SH-SY5Y cells. Knockdown efficiency was evaluated by assessing *MECP2 *mRNA (qRT-PCR) and protein (western blot analysis) expression. Time course experiments were conducted to evaluate the level and duration of the knockdown (Figure 3a and 3b). mRNA expression was consistently reduced by 75% to 80% with the greatest reduction observed at Day 3 - 4. Protein expression was consistently lowest at Day 3 - 4 with greater than 90% knockdown, and with complete recovery to normal levels between Day 7 and Day 14.

*CO1*, *CLU*, *DNMI *and *GNBI *were amongst the transcripts of interest that had statistically significant differential expression in the RTT frontal cortex. *CLU *and *CO1 *were of interest because of known associations with neurological disorders, and *CO1 *because of the described mitochondrial abnormalities observed in RTT. *DNMI *and *GNBI *were of interest because of their respective roles in endocytosis and signal transduction in neurons.

**Figure 4:**
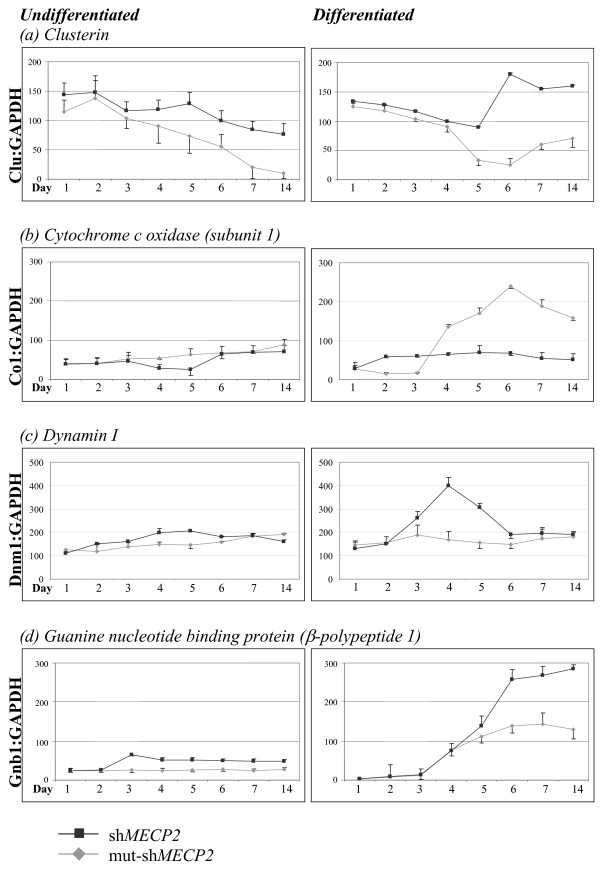
**Expression of *CO1*, *CLU*, *DNMI *and *GNBI in vitro *following RNAi-mediated knockdown of MECP2**. mRNA expression levels of selected genes of interest were assessed following RNAi-mediated knockdown of MECP2. Expression levels were evaluated in SH-SY5Y cells with or without RA-induced differentiation over a 14-day period. Expression levels of genes of interest were compared to that of *GAPDH *mRNA expression in the same sample. (a) Clusterin expression levels were higher in MECP2 knockdown samples than in controls in both differentiated and undifferentiated cells. This was more pronounced in differentiated cells where an increase in clusterin expression above basal levels was observed; (b) Cytochrome c oxidase (subunit 1) levels remained at basal levels following MECP2 knockdown in both undifferentiated and differentiated cells, whereas there was approximately a two hundred fold increase in *CO1 *expression levels in the control cells; (c) Dynamin I shows an increase in expression levels following MECP2 knockdown at Day 4, however this returned to basal levels at Day 6; (d) An increase in Guanine nucleotide binding protein β-polypeptide was observed in differentiated cells in both the MECP2 knockdown sample and the control sample, however this was 2-fold greater in the MECP2 knockdown sample.

The differential expression of these genes in RTT could be the result of a direct interaction of MeCP2 with these genes or it could be due to the interaction of MeCP2 with a gene upstream in a common pathway. We investigated the expression of these genes following RNAi-mediated knockdown of MeCP2 in SH-SY5Y cells. The expression pattern of these genes was analyzed over a fourteen day period, either with or without retinoic acid-induced differentiation of SH-SY5Y cells (Figure 4). Time course experiments were conducted twice and qRT-PCR measurement of the expression of each gene was conducted in triplicate on two occasions for each time course experiment. The expression levels of each gene were normalized to the expression levels of *GAPDH*, and therefore values on the Y axis reflect a ratio of expression of the gene of interest to the expression of *GAPDH *in the same sample. The expression of *GAPDH *is unaffected by MeCP2 knockdown (data not shown).

*CLU *mRNA expression in undifferentiated cells gradually fell throughout the 14 days (Figure 4a). The reduction was less pronounced in the MeCP2 knockdown sample however (1.9-fold reduction), than in the control (11.5-fold reduction). In the differentiated samples *CLU *expression increased in the MeCP2 knockdown sample (1.4-fold), whilst it fell in the control sample (5-fold). Therefore, the absence of MeCP2 in differentiated cells caused an uncharacteristic increase in CLU expression. It is not entirely clear why *CLU *showed a general trend downward in undifferentiated cells. Expression in SH-SY5Y cells was evaluated a number of times and for up to 21 days (data not shown). It repeatedly showed a downward trend which would plateau after 14 DIV. As clusterin is a secreted protein that binds membranes and possibly inhibits apoptosis by interfering with BAX activation in mitochondria [23], it is possible that the initial higher levels of expression were a consequence of the *in vitro *culturing of the cells and the initial stress on the cells following passaging, whilst they adhere to the culture dish and become confluent.

Undifferentiated cells showed a gradual increase in *CO1 *mRNA expression in control samples (2.2-fold by Day 7, Figure 4b). Following MeCP2 knockdown however, there was a 1.5-fold reduction of *CO1 *expression at Day 4 and Day 5. Differentiation of SH-SY5Y cells caused a dramatic rise in *CO1 *expression in the control sample (8.7-fold), however this was not reflected in MeCP2 knockdown sample, where by Day 4 *CO1 *expression increased only 2.4-fold. Thus, it appeared that a normal increase in *CO1 *expression during cellular differentiation was suppressed by MeCP2 knockdown.

*DNMI *mRNA expression remained steady in both the differentiated and undifferentiated control samples (Figure 4c). However, following MeCP2 knockdown, there was a 2-fold increase in the undifferentiated sample (Day 4) and 3-fold in the differentiated sample. In both cases expression levels returned to basal levels by Day 7.

*GNBI *mRNA expression remained steady in the undifferentiated control sample and then increased 46-fold following differentiation (Figure 4d). Following MeCP2 knockdown, *GNBI *expression increased 2.6-fold in undifferentiated samples and 77-fold in differentiated samples. Therefore, a significant increase in *GNBI *expression was observed in the absence of MeCP2.

Each of these genes showed differential expression following *in vitro *MeCP2 knockdown in the same direction (up or down regulated) observed in the patient brain samples. This differential expression was more pronounced following the differentiation of the SH-SY5Y cells into a neuron-like phenotype. These results suggest that the abnormal expression of *CLU, CO1, DNMI *and *GNBI *is due to a lack of MeCP2.

### MeCP2 associates with promoter regions of CLU, CRMP1, DNMI and GNBI

**Figure 5:**
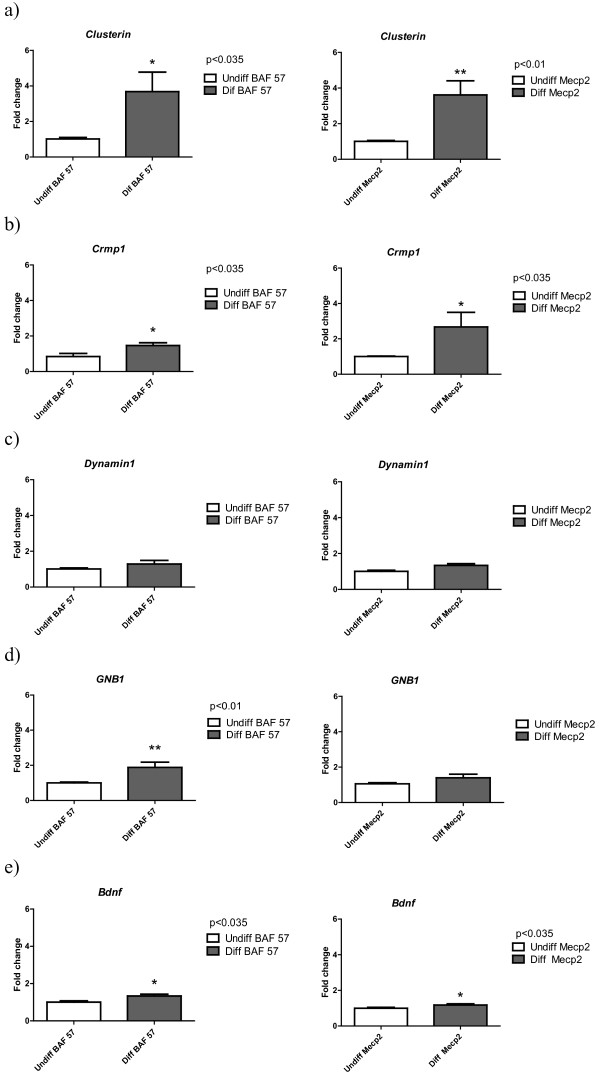
**Chromatin immunoprecipitation of MECP2**. The association of MECP2 with the promoter regions of *BDNF, CRMP1, DNMI*, *GNBI *and *CLU *in undifferentiated and differentiated SH-SY5Y cells. The *DNMI *and *GNBI *promoter sequences displayed no difference in binding with MeCP2 in both undifferentiated and differentiated cells. Results are the mean +/- SEM of six replicates. The ChIP experiments were normalized to the input DNA.

The upregulation of *CLU*, *DNMI*, *GNBI *and in some analyses, *CRMP1*, was further investigated by evaluating a potential association between MeCP2 and sequences within the promoters of these genes. *CO1 *was not investigated in this experiment due to the fact that mitochondrially-encoded genes lack CpG methylation, and it is therefore not likely to be under the direct transcriptional control of MeCP2. For differentiated and undifferentiated SH-SY5Y, proteins were cross-linked to genomic DNA, followed by shearing of the chromatin, and immunoprecipitation with an antibody specific for MeCP2 and BAF 57. After reversing the protein/DNA cross-links, the immunoprecipitated DNA was purified and subjected to quantitative real time PCR. Clusterin and CRMP1 interestingly showed minimal association with MeCP2 in undifferentiated cells. However, following RA-induced differentiation of the cells to a mature neuron-like phenotype, MeCP2 showed greater association with these genes (Figure 5a and 5b). In contrast there was a strong association between *DNMI *and *GNBI *promoter regions with MeCP2 (Figure 5c and 5d) in differentiated and undifferentiated cells. The ChIP assay clearly shows that MeCP2 is associated with *BDNF *promoter in Human SH-SY5Y cells (Figure 5e).

### Cytochrome c oxidase activity is reduced following MeCP2 knockdown

Down-regulation of *CO1 *expression in both the RTT frontal cortex postmortem tissue and *in vitro *following MeCP2 knockdown suggests mitochondrial dysfunction may contribute to RTT neuropathology. This gene is of specific interest because of reports of mitochondrial abnormalities in RTT [24-29]. To determine if down-regulation of the mitochondrially-encoded, largest subunit of cytochrome c oxidase, *CO1*, was associated with a functional abnormality of cytochrome c oxidase (COX), COX enzyme activity was assayed by measuring the rate of oxidation of reduced cytochrome c following *in vitro *MeCP2 knockdown in SH-SY5Y cell line three days post-transfection with the *MECP2 *dsRNA knockdown oligonucleotide, or the control.

**Figure 6:**
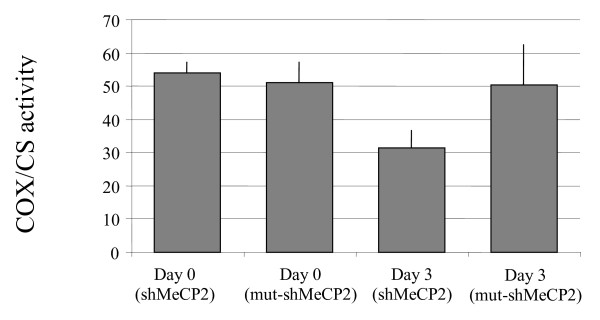
**Cytochrome c oxidase activity following RNAi-mediated knockdown of MECP2**. Cytochrome c oxidase (COX) activity was determined as a first order rate constant and expressed as rate/minute/mg protein, whilst the specific activity of the mitochondrial matrix enzyme citrate synthase (CS) was determined and expressed as nmol/minute/mg protein. COX was normalized to CS activity to take into consideration any variation in mitochondrial numbers, and is expressed as a ratio. Three days post-transfection, when MECP2 knockdown is greatest, cytochrome c oxidase activity is just 60% of that observed in the untreated sample at Day 0 (p = 0.005) and the control sample at Day 3 (p = 0.015). KD = knockdown.

The reduction in cytochrome c oxidase activity at Day 3 in the *MECP2*-deficient system (Figure 6) was statistically significant, being approximately 60% of that observed in the untreated sample at Day 0 and the control sample at Day 3. This therefore suggests that the down-regulation of *CO1 *in the frontal cortex of RTT brains may be functionally significant, as COX activity is reduced, which potentially could impact on the efficiency of ATP generation in the brain.

## Discussion

The discovery that mutations in *MECP2 *are responsible for most cases of RTT has led researchers to search for transcriptional targets and misregulated expression of genes. A profile of aberrantly expressed genes or classes of genes has slowly emerged, and subsequent downstream functional analyses of these genes are shedding light on the impact that their abnormal expression has on the pathology of RTT. Various mRNA microarray or differential display studies in human postmortem tissue [15], *Mecp2*-mutant mice [9,19-22,30], patient fibroblast [18], lymphoblastoid [16,18] and lymphocyte cells [17], and neuroblastoma (SH-SY5Y) cells in which *MECP2 *has been knocked down [30], have revealed that MeCP2 transcriptionally controls a wide range of functionally distinct genes.

Our aim was to expand the repertoire of genes that are aberrantly expressed in the RTT brain. To achieve this, we took the novel approach of evaluating differential mRNA expression between an affected and an unaffected brain region in *MECP2 *mutation-positive RTT patient postmortem tissue samples while also comparing mRNA expression in diseased and healthy tissue. We acknowledge that a potential limitation of our study is that at the time of this study we were unable to obtain equivalent age-matched control samples. It could be argued that the observed differences are a consequence of the developmental and aging processes or could be an epiphenomenon as a result of the disease process. However, we feel that the conclusions drawn in this exploratory phase of the study were valid for several reasons. Firstly, we applied very stringent criteria in selecting genes for further study, including the requirement that for a gene to be flagged as potentially significant it had to be differentially expressed in at least 5 out of the 6 relevant microarray data sets. Secondly, comparison of microarray data sets of different regions in the same subject yielded results that were consistent with our central hypothesis, namely that brain region-specific differences in gene expression would allow us to identify genes of potential biological relevance to the RTT neurodevelopmental phenotype. Thirdly, the subset of genes we chose for further analysis all showed differential expression in our RNAi-mediated knockdown system in the same direction as was seen in the microarray experiments. Finally, enzymatic studies of cytochrome c oxidase in the RNAi treated SH-SY5Y cells confirmed that the abnormalities seen at the RNA level were of functional significance at the protein level.

Only one other microarray study has used RTT patient brain samples as the source material [15]. There were, however, a number of limitations of this study, including difficulties in comparing expression data obtained by using different microarray procedures, which differed significantly in four ways - substrates (eg nylon filters versus glass slides), types of labels (radioactivity versus fluorophores), numbers of cDNA clones (eg 597 clones for one type of array versus ~7000 clones for another), and the threshold for delineating differential expression. For our studies we used only one array platform, containing around 19,000 transcripts, and employed very stringent filtering criteria with the aim of having a low false discovery rate of less than 0.6%. Using this approach we identified only a small number of genes showing altered expression in the frontal cortex of RTT patients, and our *in vitro *studies of a subset of these genes recapitulated our microarray results.

Recognized, but little studied, findings in RTT patients are morphological abnormalities of mitochondria [24,25,27], functional defects of the mitochondrial respiratory chain [26,29], and evidence of increased oxidative stress [28]. In addition, some of the non-neurological manifestations of RTT have been seen in patients with primary respiratory chain disorders, including short stature, myopathy, cardiac arrhythmias, and intestinal pseudo-obstruction [31]. However, the contribution of these functional abnormalities of the respiratory chain to the pathogenesis of RTT remains unclear. At a molecular level, an association between MeCP2 and the promoter of ubiquinol-cytochrome c reductase core protein 1 has been demonstrated (*Uqcrc1*) with subsequent up-regulation of this gene and mitochondrial respiratory chain dysfunction in *Mecp2*-null mice [21], as noted above. Here we show that cytochrome c oxidase subunit 1 expression was down-regulated exclusively in the frontal cortex of the RTT brain. This down-regulation was a consequence of MeCP2 deficiency (by RNAi analysis) and manifested as a decrease in COX enzyme activity. These two findings suggest that a defect in MeCP2 activity is likely to result in significant dysfunction of the mitochondrial respiratory chain in the brain and possibly other organs, which could contribute to the clinical abnormalities seen in RTT patients. It should be noted however that CpG methylation is absent in mitochondrially-encoded genes. Therefore, it is not likely that cytochrome c oxidase subunit 1 is under the direct transcriptional control of MeCP2, but may be affected as a consequence of a direct interaction of MeCP2 and a nuclear-encoded component of the processes responsible for mitochondrial DNA replication, transcription or translation, or direct interaction of MeCP2 with a nuclear-encoded protein that plays a role in maintaining the structure and stability of the COX protein complex.

Dynamin I was upregulated in the frontal cortex of the RTT brain, and we have demonstrated *in vitro *that it was upregulated as a result of MeCP2 knockdown, and that the promoter region of dynamin I associated with MeCP2. Dynamin I is a large GTPase that is involved synaptic vesicle endocytosis. Specifically, it assembles around the invaginated clathrin-coated pit and pinches the vesicle from the plasma membrane [32]. Other indications of abnormalities in synaptic transmission in RTT have come from studies investigating the aberrant expression of glutamate receptors such as NMDA and AMPA receptors at excitatory synapses [33,34], and associated impairment of both long term potentiation (LTP) and depression (LTD) [34], and by the correction of abnormalities in LTP when Mecp2 deficiency is reversed in a mouse model of RTT [35]. NMDA receptor expression is decreased in RTT and interestingly, the internalization of this receptor is clathrin-mediated and is facilitated by a postsynaptic member of the dynamin family, dynamin 2 [36]. Additionally, it has been demonstrated that synaptic activity can induce phosphorylation of MeCP2 which subsequently leads to gene transcription [37]. The increase in the expression of dynamin I in the frontal cortex of RTT patients may manifest as abnormal rates of synaptic vesicle cycling which adds another dimension to the idea that anomalous synaptic transmission is contributing to the severe cognitive impairment in RTT.

The regulation of expression and function of the glycoprotein clusterin remains incompletely defined. It is associated with apoptosis, and whilst it is thought to promote tumor progression in cancer through Bax interaction [23], it possibly suppresses beta-amyloid deposition in Alzheimer disease [38]. Upregulation of clusterin expression is not only associated with amyloid deposits in Alzheimer disease [39] but also with various other neurological disorders. Increased expression is associated with deposition of prion protein in transmissible spongiform encephalopathies [40], with status epilepticus [41] and following cerebral ischemia where it is associated with tissue remodeling [42]. In the brain it is expressed in specific neuronal populations of cells in the hippocampus and cerebellum. These are regions of the brain that have been implicated in dysfunction in RTT at a histological level, and increasing evidence now also suggests that these regions are also dysfunctional at a molecular level. In this study, clusterin was upregulated in the frontal cortex of RTT brains and this upregulation was the result of MeCP2 deficiency as demonstrated by *in vitro *RNAi-mediated knockdown of MeCP2. In addition, the CpG-rich promoter region of clusterin associates with MeCP2, and it is interesting to note that it has previously been shown that expression of clusterin is induced by histone deacetylase inhibitors [43], raising the possibility that one mechanism by which MeCP2 mutations may exert their effect could be by failing to repress transcription through the interaction of MeCP2 with histone deacetylase. Interestingly, in the Delgado study, clusterin was underexpressed in mutant T-lymphocyte clones from RTT patients [17]. Whether abnormal expression of clusterin is related to a pro- or anti-apoptotic function is unknown, but the fact that it is upregulated in a region that displays a greater level of cellular pathology makes it seem likely that its role in RTT is not of a neuroprotective nature.

CRMP1 is involved in semaphorin-induced growth cone collapse during neural development. It was upregulated in the RTT frontal cortex and we showed that MeCP2 associated with its promoter region in differentiated neuronal cell cultures. In adult mice, CRMP1 is localized to dendrites of cortical and hippocampal (CA1) pyramidal neurons [44], where it is possibly involved in neurite outgrowth and LTP [45]. *CRMP1 *knockout mice display abnormal MAP2 staining [45]. Abnormalities in *CRMP1 *expression in this study may explain in part the morphological abnormalities observed in neurons in the RTT brain whereby there is a decrease in dendritic arborization.

This study utilized stringent inclusion criteria for differentially expressed genes. It can be noted however (Table 1) that there was a large number of transcripts that were differentially expressed in some comparisons, but not in others. It is interesting to note that a number of the genes that were up- or downregulated in the frontal cortex were also up- or downregulated in other studies. Another component of the respiratory chain succinate dehydrogenase (subunit B), EH-domain containing 1 (endocytosis of IGF1 receptors) and amyloid precursor protein were all differentially expressed in some of the analyses in this study as well as in the study conducted by Colantuoni et al. [15]. Genes that were statistically upregulated in this study (yet didn't have a significant fold change value) were also upregulated in the Delgardo study including eukaryotic translation initiation factor 2 subunit 1 alpha, thioredoxin, autism susceptibility candidate 2 and COX 15 homolog. MIR (Membrane-associated ring finger) is a protein linking membrane proteins to the actin cytoskeleton, PAM (Peptidylglycine alpha-amidating monooxygenase) catalyzes neuroendocrine peptides and FHL1 (four and a half LIM domains) involved in cell growth and differentiation. These transcripts were all differentially expressed in the frontal cortex in this study (SAM analysis) and in RTT-derived lymphoblastoid cell lines [16]. Additionally, creatine kinase was upregulated by SAM analysis in the frontal cortex in this study and in the olfactory proteomic study [46].

This study has looked at the differential expression of genes in an area of the RTT brain that displays abnormal neuronal development. We have found that this differential expression was reproduced in an *in vitro *MeCP2 RNAi system. The promoter regions of two of these genes (*DNMI *and *GNBI*) associated with MeCP2 in differentiated and undifferentiated neuron-like cells, whilst two other genes (*CRMP1 *and *CLU*) associated with MeCP2 in differentiated cells only. In addition, downregulation of *CO1 *expression manifested as a downregulation of COX activity of the mitochondrial respiratory chain.

In summary, this study has further contributed to our understanding of how MeCP2-mediated misregulation of transcriptional repression of subsets of genes may influence the pathology in RTT. Of particular importance is the emerging role of mitochondrial dysfunction as well as the possibility that specific abnormalities in synaptic transmission contribute to the disease.

In considering the role of MeCP2-regulated genes highlighted in this study, further investigations of the effect of MeCP2 on apoptosis and/or neuroprotection, respiratory chain function, vesicle-mediated endocytosis, and secondary messenger/cell signaling in the RTT brain may provide further insight in to the pathophysiology of RTT.

## Conclusion

In this study we have used microarray technology to identify targets of *MECP2*, the gene responsible for most cases of the devastating neurodevelopmental disorder Rett syndrome. This is only the second time that brain samples from humans with Rett syndrome have been used in such a study. Using a stringent filtering approach we have found only a small number of genes show consistent altered expression in the frontal cortex of Rett syndrome patients. We provide evidence that Rett syndrome may in part be due to abnormalities of mitochondrial respiratory chain function and neuronal vesicle dynamics.

## Methods

### Patient Samples

Postmortem neural tissue from RTT and gender matched normal control individuals (Table 3) was obtained from The Harvard Brain Tissue Resource Center, Boston, USA; the New South Wales Tissue Resource Centre, Sydney, Australia; Baylor College of Medicine, Houston, USA, and The Telethon Institute for Child Health Research, Perth, Australia. All specimens were retained and used for research with appropriate written consent from the families. This research was approved by the Ethics Committee of the Children's Hospital at Westmead.

**Table 3:** Characteristics of the RTT patient and control brain samples used in this study.

Sample	Source	UId	Sex	Age	Mutation			
					**nt change**	**aa change**	**domain affected**	**type of mutation**

**RTT1**	HBTRC	4315	F	11	c.763C>T	R255X	TRD-NLS	Nonsense

**RTT3**	HBTRC	4422	F	12	c.808C>T	R270X	TRD-NLS	Nonsense

**RTT4**	TICHR	NB	F	18	c.473C>T	T158M	MBD	Missense

**RTT5**	TICHR	KB	F	11	c.316C>T	R106W	MBD	Missense

**RTT6**	TICHR	BC	F	21	c.808C>T	R270X	TRD-NLS	Nonsense

**RTT9**	BCM	93-244	F	4	c.750insC	P251fs	TRD	Frameshift/Truncation

**CON4**	NSWTRC	88210	F	43				

**CON5**	NSWTRC	88295	F	42				

**CON6**	NSWTRC	88365	F	31				

**CON7**	NSWTRC	88304	F	43				

**CON8**	NSWTRC	9092	F	46				

**CON10**	NSWTRC	12862	F	52				

Abbreviations: HBTRC = The Harvard Brain Tissue Resource Center, Boston, USA; NSWTRC = The New South Wales Tissue Resource Centre, Sydney, Australia; BCM = Baylor College of Medicine, Houston, USA; TICHR = Telethon Institute for Child Health Research, Perth, Australia; UId = unique identifier from original source; MBD = methyl-binding domain; TRD = transcription repression domain; NLS = nuclear localization signal.

None of the RTT or control patients suffered prolonged agonal states, such as prolonged premortem anoxic injury, multi-organ failure or coma. RNA integrity was assessed in nine RTT patient samples and seven were deemed to have suitably intact RNA for further analysis. One patient did not have a *MECP2 *mutation/large deletion and was omitted from the study. Samples from The Harvard Brain Tissue Resource Center, the New South Wales Tissue Resource Centre and the Telethon Institute for Child Health Research were supplied as full thickness cortical layer. In two instances we received larger coronal samples from the Baylor College of Medicine. In these instances, the outer cortical layers were isolated so that results could be comparable to the other samples. The results of the initial microarray experiments would therefore represent contributions from various outer cortical layers, and teasing out the subtleties of the contribution of individual layers would have been lost in this experiment. However, the expression changes that were observed can be concluded to be an average of these outermost cortical layers.

### Microarray studies

Four types of comparisons were made between: (1) the frontal and occipital cortices of each RTT brain; (2) RTT frontal cortices and control frontal cortices; (3) frontal and occipital cortices of each control brain, and (4) RTT occipital cortices and control occipital cortices.

All microarray experimental details were documented according to MIAME guidelines and are documented at MiameExpress (Accession number: E-MEXP-2588) [47]. Microarrays were sourced from the University Health Network (UHN) Microarray Centre, Ontario, Canada http://www.microarrays.ca. They were spotted microarrays containing 19,008 characterized and unknown sequence-verified human expressed sequence tags (ESTs), from I.M.A.G.E. Consortium, printed onto two Corning^® ^Gamma Amino Propyl Silane II (CMT-GAPS) glass slides.

Total RNA was extracted from 100 mg of postmortem brain tissue using Tri Reagent™ (Sigma-Aldrich Inc), following the manufacturer's recommended protocol. The integrity of RNA was assessed by running approximately 1 μg of total RNA on a 1.2% (v/v) formaldehyde agarose (FA) gel. Messenger RNA was amplified from total RNA using a modified version of the Eberwine ("antisense") RNA amplification protocol [48,49]. First strand cDNA synthesis from total RNA was conducted using an oligo d(T)/T7 primer and SuperScript™ II RNase H^- ^Reverse Transcriptase (Invitrogen™ Life Technologies). Second strand synthesis was conducted using *E. coli *DNA Polymerase I and RNaseH (Invitrogen™ Life Technologies). Linear *in vitro *transcription of mRNA was undertaken using the MEGAscript™ High Yield Transcription Kit (Ambion^®^) according to the manufacturer's instructions. Amplified mRNA was reverse transcribed and a modified nucleotide (amino allyl-dUTP) was incorporated in the following ratio: 25 mM dATP, 25 mM dCTP, 25 mM dGTP, 15 mM dTTP, 10 mM aa-dUTP. The aa-labelled cDNA was fluorescently labeled with cyanine 3 (Cy3) or cyanine 5 (Cy5) monofunctional reactive dyes (Amersham Biosciences Pty Ltd). For subsequent hybridization, the total number of picomoles of dye incorporated for each sample was greater than 200 picomoles in a ratio of less than 80 nucleotides/dye molecules. Microarrays were scanned on an Axon GenePix^® ^4000B laser scanner (Axon Instruments).

Data normalization and partial analysis were carried out using the Bioconductor libraries of R http://cran.us.r-project.org/. The raw data from each group of experiments was normalized using a smoothing function, Loess in the R open-source software package.

Two approaches were taken in order to most accurately determine differential mRNA expression:

(1) The average fold change was a measure of the relative expression of the test sample to the control sample. This was evaluated after a number of stringency criteria were applied to the data set. Relative hybridization of the two samples being analyzed had to result in a normalization factor of the ratio of the medians between 0.8 and 1.2. All flagged features were removed prior to analysis. In addition, the absolute hybridization value in *either *the red (F635) or green (F532) channels for each spot/feature was required to exhibit a minimum intensity of 200. To determine fold change the expression level of each gene in the sample was divided by the expression level of the same gene in the control. Any gene with a value of >1.5 or <0.67 was regarded as differentially expressed. This level of fold change (1.5) was previously determined by the following: two aliquots of the same batch of RNA from a postmortem control brain sample were amplified, reverse transcribed, labeled, hybridized and normalized as described above. The standard deviation of the data was calculated to be 0.25, which was interpreted as a 95% confidence level that data with a fold change greater than 1.5 were likely to represent a true change. Six independent replicate brain tissues were evaluated. A gene that was differentially expressed in at least 5 microarray data sets was considered differentially expressed.

(2) Significance Analysis of Microarrays (SAM): SAM was used to statistically assess expression changes for each gene (*i*). The test provides a score (*d*_*I*_) based on the standard deviation of repeated permutations of the data http://www.utulsa.edu/microarray/Articles/sam manual.pdf. Data from microarray experiments were assessed in relation to a response variable (for example, RTT vs. control). The score generated was indicative of the strength of the relationship between mRNA expression and the response variable. A threshold for significance is denoted as delta, which is arbitrarily set based on a desired false positive rate (0.05).

### Quantitative Reverse Transcription (qRT-PCR)

The expression of genes that were identified in the microarray experiments as being up or downregulated were validated by qRT-PCR using a Rotor-Gene™ 3000 Real Time Thermal Cycler. A separate RNA extraction from each of the same postmortem samples was performed as previously described (this RNA was not linearly amplified as it was for the microarray experiments). Each gene of interest was amplified using primers that spanned an intron and lay close to the 3' end of the gene. Primers and PCR conditions can be provided on request. mRNA expression levels of genes of interest were evaluated in both the frontal and occipital cortex of each RTT and control sample, and normalized to *GAPDH *expression from the same sample. Each sample was evaluated in triplicate within an experiment and three independent experiments were conducted. A Mann-Whitney U test was used to test for significance.

### *In vitro* RNA interference (RNAi)

Human SH-SY5Y neuroblastoma cells (ATCC^® ^CRL-2266™) were grown to 90% confluence in a 24-well plate format in D-MEM/F-12 and FBS (Gibco^®^) at 37°C/5% CO_2_.

A dsRNA transcript that targeted a 21 base pair sequence in exon 3 of *MECP2 *(shMeCP2; AAGCATGAGCCCGTGCAGCCA; nucleotides 291-311; **NM_004992**) was used to knockdown MeCP2 in SH-SY5Y cells. This sequence is present in both the *MECP2_*e1 and e2 isoforms. The negative (non-silencing) control RNA transcript targeted a 21 base pair sequence (mut-shMeCP2; AATTCTCCGAACGTGT CACGT) that had no homology to the human genome (Qiagen Pty Ltd).

dsRNA was transfected into SH-SY5Y cells at a 1:6 ratio of dsRNA:RNAiFect transfection reagent (Qiagen). After 3 days, MeCP2 expression/knockdown was evaluated by qPCR and Western blot analysis. Cells were differentiated with 10 μM retinoic acid (RA). Cells were lysed in: 400 mM KCl, 50 mM HEPES, 1.5 mM EDTA, 20% (v/v) glycerol, 0.5% (v/v) NP40, 20 mM sodium fluoride (NaF), 10 mM sodium molybdate, 100 μM sodium ortho-vanadate, and 1 mM dithiothreitol and protease inhibitors, (1 mM phenyl-methy-sulphonyl fluoride [PMSF], 0.2 mg/mL Bacitracin, 0.2 mg/mL Aprotinin, 5 μg/mL Leupeptin and 5 μg/mL pepstatin A). Fifty micrograms of total protein was run on duplicate 8% SDS-PAGE gels and immunoblotted with either mouse anti-actin (1:10,000; kindly donated by Oncology Research Unit, The Children's Hospital at Westmead) or rabbit anti-MeCP2 (1:500; Peptide: Auspep, Antibody: Strategic Biosolutions). Primary antibodies were detected with HRP-conjugated sheep anti-mouse (1:2000; Amersham Biosciences Pty Ltd) or goat anti-rabbit (1:2000; Santa Cruz) and chemiluminescence. The film was scanned on a BioRad GS-800 Calibrated densitometer (BioRad Laboratories) and Quantity One^® ^4.2.2 software was used to semi-quantitate bands on the Western blot.

### Chromatin Immunoprecipitation

ChIP analysis was performed following the instructions recommended by the supplier (Upstate Biotechnology) with some modifications. Briefly, proteins were cross-linked to DNA by adding formaldehyde to a final concentration of 1%. 2.5 M Glycine at a final concentration of 125 mM was added to quench formaldehyde cross-linking. Samples were resuspended in SDS lysis buffer (1% SDS, 10 mM EDTA, 50 mM Tris-HCl, pH 8.1, Roche Complete Protease Inhibitor tablet) and sonicated for 15 min, using 30 sec on/off cycle (Diagenode sonicator) to shear the chromatin. The size of the genomic fragments after sonication was between 200-500 bp. The soluble chromatin fraction was incubated overnight at 4°C rotating, with either: 5 μg anti-MeCP2 [donated by Dr. Peter L. Jones], Mecp2 (9317 Sigma), 5 μg BAF 57 (donated by Dr Said Sif) and 5 μg rabbit IgG (Santa Cruz) as a negative control. Immune complexes were collected with protein A/G agarose beads and the supernatant fraction kept as the unbound DNA control. The chromatin-antibody complex was washed with low salt buffer (0.1% SDS, 1% Triton X-100, 2 mM EDTA, 20 mM Tris-HCl pH 8.1 and 150 mM NaCl), followed by washes in high salt buffer (0.1% SDS, 1% TritonX-100, 2 mM EDTA, 20 mM Tris-HCl pH 8.1 and 500 mM NaCl), lithium chloride buffer (0.25 M LiCl, 1% NP-40, 1% deoxycholate, 1 mM EDTA and 10 mM Tris-HCl, pH 8.1) and two washes in TE Buffer (10 mM Tris-HCl, 1 mM EDTA, pH 8.0). The chromatin-bead conjugate was resuspended in freshly prepared elution buffer (1% SDS, 0.1 M NaHCO_3_) and incubated at room temperature, rotating for 30 minutes. The supernatant fraction was collected and NaCl added to final concentration of 100 mM before phenol/chloroform extraction. Crosslinks were reversed by incubating at 65°C overnight. Samples were recovered by phenol/chloroform extraction and ethanol precipitation. Specific primers used for PCR were:

*Clusterin*: 5'-GTGGCGCTTGTGTAATGTGAA, 3'-TCACCACGAATAGCTGTGCTG;

*CRMP1*: 5'-GCTGGTTCAATGCTAGGATGG, 3'-ACGTTCTTGTCCCTCCAGGAT

*Dynamin1*: 5'-AGGAAGCCCATCTGCTCTCC, 3'-GGGCATCATGGGTGTCGTAG

*GNB1*: 5'-CGGAACTCAGCTGGAAAGACA, 3'-AACGAAGTCAAGAAGGCCACA

*BDNF*: 5'-AGCCCAACAACTTTCCCTT, 3'-GAGAGCTCGGCTTACACAGG

### Quantitative Real Time PCR

Quantitation of DNA extracted from three independent ChIP experiments was done by real time PCR using primers targeting the promoter regions of the following genes - *BDNF*, *CRMP1*, *Clusterin*, *GNB1 *and *Dynamin*1. The data were analyzed by the 7500 Fast Software (Applied Biosystems). Quantification was performed using the comparative CT method and is reported as the *n*-fold difference in antibody bound chromatin normalized against the input DNA (Figure 5).

### Cytochrome c oxidase assays

The method used for assaying cytochrome c oxidase activity (COX) has been previously described [50]. COX activity was determined as a first order rate constant and expressed as rate/minute/mg protein. The assay was performed at 550 nm and follows the decrease in absorbance resulting from the oxidation of reduced cytochrome c after addition of sample. Reduced cytochrome c was prepared as per Trounce *et al. *[51].

Citrate synthase is a nuclear encoded enzyme of the tricarboxylic acid (TCA) cycle, located in the mitochondrial matrix. Its activity was assayed and used to normalize measured respiratory chain enzyme activity [51].

Mann-Whitney U non-parametric analysis was used to determine the statistical significance in COX and citrate synthase enzyme differences between MeCP2 knockdown and control cells.

## Authors' contributions

JHG performed microarrays, quantitative PCR, RNA interference and cytochrome c oxidase assays, BS and JC conceived the study and participated in its design and coordination and edited the manuscript, HKN and AE-O performed the ChIP assays, SLW maintained cell lines and assisted with ChIP assays, DM performed cytochrome c oxidase assays, JLS helped with microarray data analysis. All authors read and approved the final manuscript.
